# Supra-Vaginal Amputation of the Uterus for Rupture

**Published:** 1889-12

**Authors:** 


### SUPRA-VAGINAL AMPUTATION OF THE UTERUS FOR RUPTURE.

We have received from Dr. Henry E. Coe, of New York, a reprint from the *Medical Record*, giving a report of a successful case of "Supra-vaginal Amputation of the Uterus for Rupture in Labor." We deem it worthy of mention, since it is the first successful case of the kind in this country. Fourteen cases have been reported, of which five recovered. The only other case in America proved fatal. The patient was operated upon in a squalid tenament house, amidst most unfavorable surroundings, without any previous preparation, and with the patient in a state of collapse. Prevot's operation was performed, and the patient was up at the end of four weeks. Dr. Coe is to be congratulated upon his boldness in operating under such circumstances, which was fully justified by the happy issue of the case.

### ITEMS.

A CORRECTION.—In New York letter of October, page 570 lines 11 and 15, "*acne cachicticorum*" is printed "*acute cachicticorum.*"

WE are in receipt of J. H. Chambers & Co.'s visiting list for 1890, which is very compact and conveniently arranged. Price, 75 cents.

MR. E. C. AMIS, agent for the N. Y. Pharmacal Association, was in town last month distributing, among the profession, samples of the excellent preparations of Lactopeptine.

DR. WM. A. HAMMOND, of Washington, D. C., has a larger number of patients in his sanitarium for the treatment of mental and nervous diseases than ever before in the history of the institution, although he opened about a year ago with more than half the rooms occupied in his immense new building.

Dr. Hammond is conducting a number of experiments in the treatment of epilepsy by localizing the brain lesion, trephining and paring the convolutions. He will publish the result of his experiments in the near future.

THE AMERICAN ASSOCIATION for the study and cure of inebriety gave a dinner to its President, Dr. Joseph Parrish, in honor of the seventy-first anniversary of his birth, at his home, Burlington, New Jersey, on Tuesday, November 12, 1889, at midday. Committee: Albert Day, M. D., Boston, Mass.; Lewis D. Mason, M. D., Brooklyn, N. Y.; Thomas D. Crothers, M.D., Secretary, Hartford, Conn.

DR. J. L. MILLER, of Due West, S. C., died at his home, October 17, 1889. He was a graduate of Erskine College, S. C., and of Jefferson Medical College. The degree of A. M. was conferred on him by Erskine College and by Jefferson Literary College of Penn. Dr. Miller was an able, conscientious, Christian physician. He passed his three score years, and be it said to his memory his life was as useful as it was long.

